# Prevalence of Anxiety Symptoms and Associated Clinical and Sociodemographic Factors in Mexican Adults Seeking Psychological Support for Grief During the COVID-19 Pandemic: A Cross-Sectional Study

**DOI:** 10.3389/fpsyt.2022.749236

**Published:** 2022-03-14

**Authors:** Alejandro Dominguez-Rodriguez, Paulina Erika Herdoiza-Arroyo, Reyna Jazmin Martínez Arriaga, Eduardo Bautista Valerio, Joaquín Mateu Mollá, Anabel de la Rosa-Gómez, Luis Farfallini, María Jesús Hernández Jiménez, Esteban Eugenio Esquivel Santoveña, Flor Rocío Ramírez-Martínez, Rosa Olimpia Castellanos Vargas, Carlos Armando Arzola-Sánchez, Paulina Arenas-Landgrave, Sofía Cristina Martínez-Luna

**Affiliations:** ^1^Health Sciences Area, Valencian International University, Valencia, Spain; ^2^School of Psychology, Universidad Internacional del Ecuador, Quito, Ecuador; ^3^Departamento de Clínicas de Salud Mental, Centro Universitario de Ciencias de la Salud, Universidad de Guadalajara, Guadalajara, Mexico; ^4^Facultad de Estudios Superiores Iztacala, Universidad Nacional Autónoma de México, Mexico City, Mexico; ^5^Facultad de Psicología, Universidad de Buenos Aires, Buenos Aires, Argentina; ^6^Departamento de Ciencias Sociales, Universidad Autónoma de Ciudad Juárez, Juarez City, Mexico; ^7^Rectoría, Universidad Autónoma de Ciudad Juárez, Juarez City, Mexico; ^8^Departamento de Ciencias de la Salud, Universidad Autónoma de Ciudad Juárez, Juarez City, Mexico; ^9^Coordinación Académica de Profesional, Universidad Tec Milenio, Juarez City, Mexico; ^10^Facultad de Psicología, Universidad Nacional Autónoma de México, Mexico City, Mexico

**Keywords:** COVID-19, grief, anxiety, depression, sleep quality, post-traumatic stress, drugs, Mexico

## Abstract

The COVID-19 pandemic is one of the greatest challenges in modern history, with more than four million confirmed deaths worldwide. To date, evidence regarding the psychological impact of the COVID-19 pandemic on grievers is scarce for developing countries such as Mexico. This study aimed to assess the levels of anxiety and associated concerns in a sample of Mexican adults bereaved during the COVID-19 outbreak. A cross-sectional study was conducted through the *Duelo COVID* (COVID Grief) platform, which is a self-guided online treatment. A total of 5,224 participants reported their anxiety, depression, sleep quality, avoidance, and arousal, prolonged grief symptoms, and medication consumption. Independent sample Mann-Whitney *U*-tests, chi-square tests, and Kruskal-Wallis tests, as well as multinomial logistic regression, were conducted. Results indicated that 90.4% of the participants reported clinical levels of anxiety, depression, and sleep affectations. The people who lost someone during the last 5 months scored higher in normal grief symptoms compared to the people whose loss was 6 months ago or more, and 9.8% of individuals reported the use of prescription medication, with anxiolytics and antidepressants being the most common. Females, younger respondents, unemployed people with a lower educational level, and participants who disclosed a recent suicide attempt were among those who reported medication consumption. Sleep problems were more frequent in older participants.

## Introduction

The 2019 coronavirus disease (COVID-19) has wreaked major havoc on people's mental health worldwide (1). Recent findings showed a significant increase in clinical disorders like anxiety, depression, post-traumatic stress disorder (PTSD), and insomnia (2). Shah et al. (3) reported that 50.9% of their adult sample suffered from anxiety, 57.4% from stress, and 58.6% from depression. Wang et al. (4) systematically reviewed 68 studies comprising 288,830 participants from 19 countries and found that 33% of adults reported symptoms of anxiety or depression. Likewise, Xiong et al. (5) conducted a systematic review in which they observed that the prevalence of anxiety symptoms ranged from 6.33 to 50.9%, and was often comorbid with depression at rates from 14.6 to 48.3%. Before the pandemic, Medina-Mora et al. (6) reported that in 2018, among 5,826 Mexican adults, the rate of anxiety disorder was 14.3%, followed by mood disorders at 9.2%.

During the pandemic, Pérez-Cano et al. (7) found that in a sample of 613 Mexican adults, 48% reported mild to severe anxiety, and 18% reported depression or moderate-to-severe stress. González-Ramírez et al. (8) examined the psychological impacts of COVID-19 prevention measures in a sample of 3,932 participants from the Mexican population, and observed that 943 participants showed intrusive thoughts, 933 avoidance, and 515 hyperarousal symptoms. The researchers also found that 1,160 participants showed symptoms of clinically significant post-traumatic stress. Avoidance responses included denial of the event's significance and consequences, blunted sensation, and awareness of emotional numbness (9). On the other hand, arousal included anger, irritability, hypervigilance, and difficulty concentrating (10).

Within these studies, predictors of anxiety, depression, stress, and PTSD included being under the age of 40, being of female gender, being divorced or widowed, having a lower education level, suffering from poorer health, feeling alone, living in urban areas (5), and having poor sleep quality (11). Wang et al. (4) identified that higher odds of anxiety and depression were related to COVID-19 infection risk that included suspected or confirmed cases, suffering from pre-existing physical or mental conditions, and longer media exposure. Likewise, it has been found that the population who contracted COVID-19 along with those with pre-existing diseases, particularly chronic/degenerative pathologies, or other psychiatric disorders were related to higher levels of anxiety and depression (12). Anxiety has also been associated with poor sleep quality in the context of the COVID-19 pandemic (13), as well as potentially increased rates of abuse of psychotropic medication and illegal drugs (14), as means to cope with suffering.

A section of the population that may be particularly impacted during the pandemic are those who have experienced a bereavement (15). According to the World Health Organization (WHO), as of July 12, 2021, there were 5,570,163 deaths due to COVID-19 since the beginning of the pandemic (1).

Mexico is the fourth highest place in terms of deaths due to COVID-19. This means there are now millions of families that are currently grieving a person who has died from COVID-19, not to mention other causes on death (16).

Experiencing a sudden loss, such as those brought on by COVID-19, may have a significant impact on those left to grieve (13). Kokou-Kpolou et al. (16) indicated that death circumstances during the pandemic due to the viral contagion of COVID-19 deprive families of assisting the dying person and partaking in associated rituals or ceremonies, provoking an increase in emotional pain and raising the likelihood of pathological grief outcomes. Other factors that might complicate the grieving process include feelings of guilt, the restrictions imposed on funerals to share emotional pain with others, and the isolation prior to the loss (17, 18), as well as anticipatory grief that could lead to mental health complications in both the surviving family and the medical staff (18).

Given these risk factors and psychological needs, the online intervention *Duelo COVID*, a self-guided treatment that provides psychological intervention aimed at preventing the development of complicated grief in the Mexican population during the COVID-19 pandemic, was created (19). This online platform was designed for those who have experienced a bereavement during the pandemic, regardless if it was due to COVID-19 or another cause during the pandemic. It consists of 12 sessions with the aim to assist participants in resolving conflicts associated with a higher risk of grief complications and to improve wellbeing in different areas of the affected people's lives. This study was carried out as a result of the data obtained from *Duelo COVID* to further our understanding of the impact of bereavement during COVID-19 on levels of psychological distress in Mexico. Specifically, the study explored levels of anxiety, depression, sleep quality, avoidance, arousal, prolonged grief symptoms (also known as complicated grief), and medication consumption with the aim of better understanding the needs of this vulnerable population.

## Methods

### Study Design and Participants

**Figure 1:**
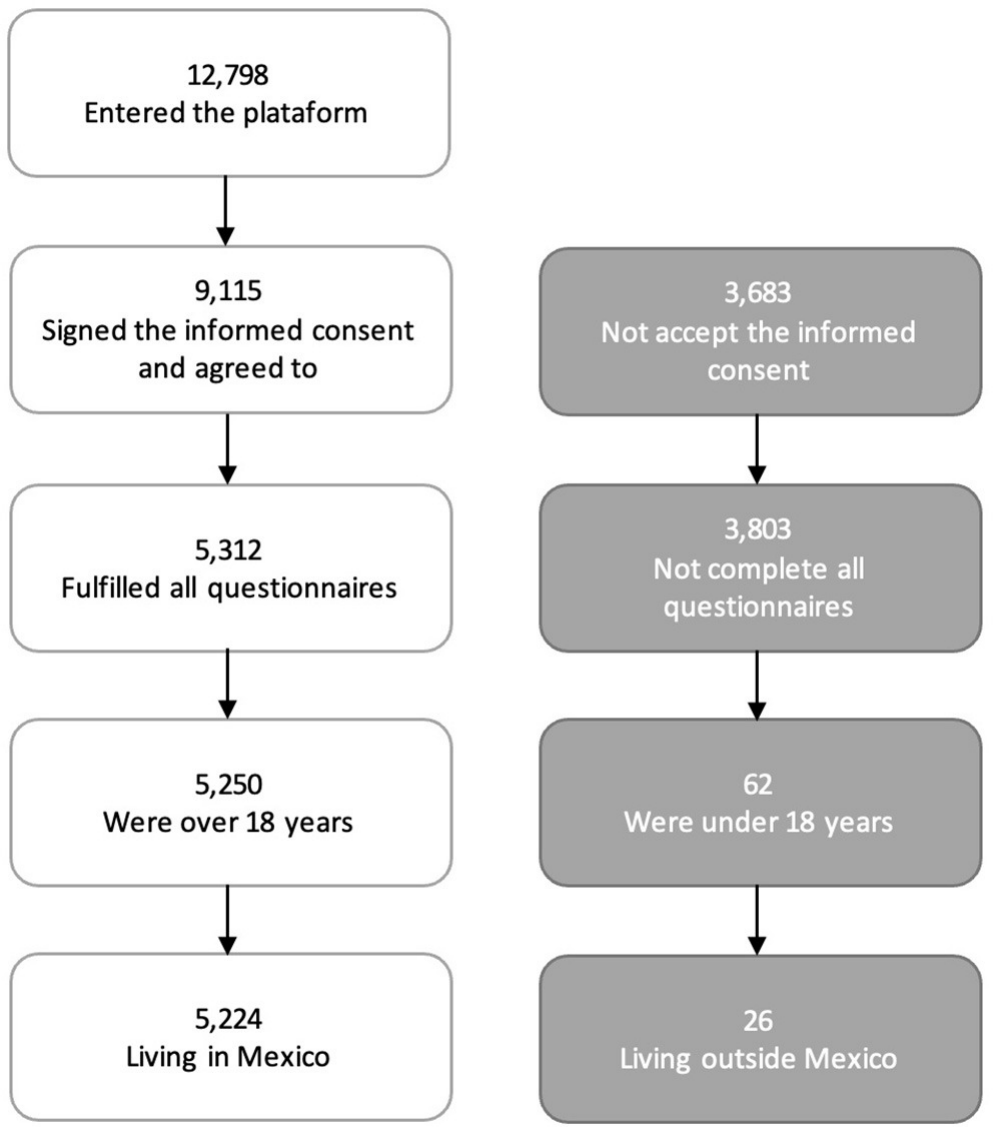
Sample filtering flow chart.

The first step in the analysis was the selection of participants who met the inclusion criteria. Using non-probability sampling and a cross-sectional design, 12,798 participants were recruited from those who had accessed *Duelo* COVID from mid-December 2020 to mid-March 2021. After excluding 3,683 individuals who had not signed the informed consent form, the included participants were those who had completed the pre-evaluation questionnaires, were aged 18 years or older, and had accessed the platform from Mexico (*N* = 5,224). Figure 1 shows the sample filtering flow chart. Sociodemographic characteristics of the final sample are presented in Table 1.

**Table 1:** Sociodemographic characteristics of support-seeking bereaved adults during the COVID-19 pandemic.

	**Anxiety**				**Depression**				**Sleep**				**Avoidance**				**Arousal**			
**(*n*; %)**	**Mdn [IQR]**	***U***	***p***	***r***	**Mdn [IQR]**	***U***	***p***	***r***	**Mdn [IQR]**	***U***	***p***	***r***	**Mdn [IQR]**	***U***	***p***	***r***	**Mdn [IQR]**	***U***	***p***	***r***
**Gender**																				
Female (4,559; 87.3)	14.00 [9.0]	1263,4 13.50	<0.001	0.13	34.00 [22.00]	1302,1 41.50	<0.001	0.11	12.00 [5.00]	1303,5 28.00	<0.001	0.11	11.00 [8.00]	1366,4 69.00	0.001	0.08	8.00 [6.00]	1239,7 24.00	<0.001	0.14
Male (654; 12.5)	12.00 [9.0]				29.00 [23.00]				11.00 [5.00]				10.00 [8.00]				7.00 [6.00]			
**Age in years**																				
≤ 30 (2,406; 46.1)	14.00 [8.00]	3057,4 41.00	<0.001	0.09	34.00 [21.00]	3109,3 38.00	<0.001	0.07	12.00 [5.00]	3127,0 07.00	<0.001	−0.09	12.00 [7.00]	2897,4 84.50	<0.001	0.13	9.00 [5.00]	3066,4 56.50	<0.001	0.08
≥31 (2,818; 53.9)	13.00 [10.00]				32.00 [23.00]				12.00 [5.00]				10.00 [8.00]				8.00 [6.00]			
**Working**																				
Yes (3,100; 59.3)	13.00 [9.00]	3111,5 41.00	0.001	−0.05	31.00 [22.00]	2882,8 09.50	<0.001	−0.11	12.00 [5.00]	3182,0 40.50	0.039	−0.03	10.00 [8.75]	3058,0 02.50	<0.001	−0.06	8.00 [6.00]	3073,3 92.00	<0.001	−0.06
No (2,124; 40.7)	14.00 [8.00]				35.00 [21.00]				12.00 [5.00]				11.00 [8.00]				9.00 [5.00]			
**Education in years**																				
≤ 12 (1,243; 23.8)	14.00 [8.00]	2316,2 43.00	0.001	0.06	37.00 [20.00]	2011,0 24.00	<0.001	0.16	12.00 [5.00]	2284,9 95.00	<0.001	0.07	11.00 [8.00]	2272,4 48.50	<0.001	0.07	9.00 [6.00]	2247,6 05.50	<0.001	0.08
≥13 (3,981; 76.2)	13.00 [9.00]				32.00 [22.00]				12.00 [5.00]				10.00 [8.00]				8.00 [6.00]			
**Psychological support**																				
Yes (402; 7.7)	14.00 [9.00]	934,21 4.00	0.897	–	31.00 [23.00]	868,31 1.50	0.013	−0.07	12.00 [6.00]	929,88 8.50	0.776	–	10.00 [8.00]	851,91 2.50	0.002	−0.08	8.00 [6.00]	892,73 2.00	0.108	–
No (4,666; 89.3)	13.50 [9.00]				33.00 [22.00]				12.00 [6.00]				11.00 [8.00]				8.00 [6.00]			
**Medications**																				
Yes (513; 9.8)	17.00 [7.00]	847,75 2.50	<0.001	0.24	39.00 [19.00]	895,47 6.50	<0.001	0.20	15.00 [5.00]	677,57 5.50	<0.001	0.36	13.00 [8.00]	981,97 5.00	<0.001	0.13	10.00 [6.00]	904,49 7.00	<0.001	0.19
No (4,555; 87.2)	13.00 [8.00]				32.00 [22.00]				12.00 [5.00]				11.00 [8.00]				8.00 [6.00]			
**Suicide attempt**																				
Yes (164; 3.14)	18.00 [6.00]	242,88 9.00	<0.001	0.35	48.00 [13.00]	169,01 0.00	<0.001	0.50	14.00 [5.00]	276,59 4.50	<0.001	0.27	15.00 [6.00]	216,17 1.50	<0.001	0.41	12.00 [5.00]	228,89 4.00	<0.001	0.38
No (4,904; 93.9)	13.00 [9.00]				32.50 [22.00]				12.00 [5.00]				11.00 [8.00]				8.00 [6.00]			
**Time since loss**																				
<6 months (4,264; 84.6	14.00 [9.00]	1702,0 76.00	0.751	–	33.00 [22.00]	1567,5 41.50	<0.001	0.08	12.00 [5.00]	1615,5 03.00	0.009	0.05	11.00 [8.00]	1703,2 15.50	0.774	–	8.00 [6.00]	161,91 3.50	0.012	−0.05
≥6 months (804; 15.4)	13.00 [9.00]				31.00 [22.00]				12.00 [5.00]				11.00 [8.75]				9.00 [6.00]			

*Mdn, Median; IQR, interquartile range; r, Rosenthal's r. Missing data: n, 11 (0.2%) for gender, and n, 156 (3.0%; the same participants) for psychological support, Medication intake, Suicide attempt, and Time since loss*.

### Psychological Measures

#### Generalized Anxiety Disorder 7-Item

The GAD-7 is a 7-item scale designed to measure the severity of symptoms of generalized anxiety disorder (20). Items range from 0 (*not at all*) to 3 (*nearly every day*), with a possible score ranging from 0 to 21. A cut-off point of 5 or above is an indicator of probable anxiety symptoms. Additional cut-off criteria were used to explore the severity of those symptoms: GAD7 ≥10 and GAD7 ≥15 for moderate and severe presentations, respectively (20, 21). In the current study, Cronbach's alpha = 0.90.

#### Center for Epidemiologic Studies Depression Scale

The CESD-R is a self-report scale that enquires about symptoms of depression during the past 2 weeks. It consists of 20 questions with answers ranging from *rarely* or *never* (<1 day) to *most of the time* (5–7 days). Scores of 16 or above indicate probable depression diagnosis (22). This measure has been commonly used in health research, and its psychometric properties have shown it to be a valid scale in the Mexican population (Cronbach's alpha >0.90) (23). In this study, Cronbach's alpha was 0.93.

#### Pittsburgh Sleep Quality Index

The PSQI assesses sleep quality patterns. Seven areas are evaluated including subjective sleep quality, sleep latency, sleep duration, habitual sleep efficiency, sleep disturbances, daytime dysfunction, and use of sleeping medication. A total score ranging from 0 to 21 is provided by the sum of the seven mentioned factors. A score of five or above indicates poor sleep quality (24). In the present study, we will report this total score. The measure has demonstrated reliability in the Mexican population (α = 0.78) (25). In the current study, Cronbach's alpha = 0.70.

#### Post-traumatic Stress Disorder Symptom Scale Self-Report

The PSS-SR is a 17-item semi-structured self-report measure that can be administered to examine post-traumatic symptoms. Each item is answered using a four-point scale ranging from 0 (*not at all*) to 3 (*very much*). The instrument presents adequate reliability values (α = 0.85) (26, 27). In the present study, two of the three dimensions of this scale were considered (due to their link with anxiety): avoidance (α = 0.82) and increased arousal/ activation (α = 0.76) of physical, cognitive, or behavioral symptoms derived from post-traumatic stress. Both will be considered for the data analysis.

#### Inventory of Complicated Grief

The ICG (28) is a self-report measure composed of 19 items that assess emotional, cognitive, or behavioral symptoms of complicated grief (CG). For this study, the Spanish version of Limonero et al. (29) was applied. Items range from 0 (*never*) to 4 (*always*), with a total score ranging from 0 to 76. A cut-off point over 30 in the total ICG score was used to explore potentially maladaptive grief symptoms. The validation of the scale in the Mexican population was performed by the authors of the present study and the preprint version is available at Dominguez-Rodriguez et al. (30) In the current study Cronbach's alpha = 0.93.

### Sociodemographic Information

Participants were asked to report their gender and, in a binary *yes/no* format, their employment status, if they were currently receiving psychological treatment, and if in the last 3 months they had attempted to take their own life. They were also asked to inform about any medications they were taking at the time of the study.

Three other socio-demographic variables were re-coded as follows: age ( ≤ 30 years old; ≥31 years old), educational level ( ≤ 12 years; ≥13 years), and time since loss (<6 months; ≥6 months). These cut-off scores were decided upon for the following reasons: the age of 30 is commonly used for separating young adults from adults. Twelve years represent the average time spent in mandatory education in the countries where the participants of this study reside. The time frame of 6 months or more, elapsed since the loss, is used to assess the presence of persistent and potentially problematic symptoms of grief, as proposed by the ICD-11 (31). Initially, these last three variables were, respectively, continuous (for age) and eight-point ordinal variables (for educational level and time since loss).

### Procedures

From December 22, 2020, to March 16, 2021, participants accessed the online platform and completed the informed consent form followed by the questionnaires. At the end of the survey, the participants were notified about available online psychological counseling services, if the users were identified as being at risk after answering the questionnaires (e.g., recent attempts of suicide, moderate to high results on the suicide scale, diagnosis of posttraumatic stress disorder), and if the participants did not have access to the intervention, they were informed about other available free psychological services. Also, one excluded participant requested their data to be removed from the data analysis.

The study design and procedures were approved by the Research Ethics Committee of the Autonomous University of Ciudad Juárez, México (Approval ID: CEI-2020-2-226) and is registered in Clinical trials (NCT04638842).

### Statistical Analysis

Data analyses were performed using SPSS statistical software version 23.0. The prevalence of anxiety, depression, sleep problems, and grief symptoms was expressed as raw scores and percentages of cases and was calculated using the following cut-off scores: for anxiety, GAD-7 ≥5; for depression, CESD-R ≥16; for sleep problems, PSQI >5, and for potentially problematic grief, ICG >30. The prevalence rates of the two post-traumatic stress factors (avoidance and activation) were not calculated, because we used only two factors (and not the full scale), and we do not have a cut-off point that allows this threshold to be established. The normality and homoscedasticity were calculated for all the dependent variables by the Kolmogorov-Smirnov and the Levene tests, respectively. Independent sample Mann-Whitney *U*-tests were used to explore the sociodemographic characteristics of the participants, including gender, age, working status, educational level, medication intake, psychological treatment, suicide attempt in the last 3 months, and time since loss of the loved one, as well as the total scores of anxiety, depression, sleep problems, avoidance and activation (arousal), and grief symptoms. Rosenthal's *r* was computed to assess the magnitude of the effect for the statistically significant differences obtained from the comparative analyses (32).

Pearson's correlations were used to explore the bivariate correlations among the continuous variables of this study (depression, sleep problems, avoidance, and increased arousal), as a first step to test multicollinearity.

A Chi-square test was performed to compare two independent groups when categorical variables were used, whereas the Kruskal Wallis test was used for comparing more than two groups in a quantitative variable. Chi-Square and Kruskal-Wallis tests, with Cramer's V and Epsilon Squared, respectively, were also performed to compare sociodemographic characteristics and clinical correlates among different groups of participants regarding anxiety severity: individuals who did not present significant anxiety symptoms (GAD-7 = 1–4), and those with mild (GAD-7 = 5–9), moderate (GAD-7 = 10–14), and severe (GAD-7 = 15–21) levels. Multinomial logistic regression was used to quantify the effects of sociodemographic variables on symptoms of anxiety. Significant associated variables identified from the chi-square tests were entered into logistic regression. The Nagelkerke-R^2^ was used to examine the percentage of variance associated with anxiety that was explained by the categorical predictors. Adjusted odds ratio with 95% confidence intervals were also reported to measure the strength of association. To identify the relationship between anxiety level and medication use, chi-square and Cramer's V were calculated.

## Results

### Clinical Symptoms and Sociodemographic Characteristics

There were 4,895 (93.7%) respondents who reported symptoms of anxiety that were rated as mild or above (GAD ≥5); 4,972 (95.2%) had clinically relevant sleep problems (PSQI >5), 4,526 (86.6%) presented mild or greater depression symptoms (CESDR ≥16). Of those, 4,724 (90.4%) were high across the three areas. Potentially problematic grief symptoms were displayed by 3,538 (67.7%) (ICG >30).

As presented in Tables 1, 2, female gender, under 30 years old, unemployed, lower educational level, taking medication, and made a recent suicide attempt were associated with higher levels of anxiety, depression, posttraumatic stress symptoms (avoidance and activation), and grief symptoms. The results were similar for sleep difficulties; however, older rather than younger age was associated with having difficulties.

**Table 2:** Levels of complicated grief symptoms by sociodemographics.

**Variable**		**Mdn (IQR)**	***U***	***p***	***r***
Gender	Female	40 (24)	1183,546.00	<0.001	−0.09
	Male	35 (26)			
Age	≤ 30 years old	41 (24)	2912,204.00	<0.001	−0.09
	≥31years old	38 (25.75)			
Work status	Employed	38 (24)	2845,588.50	<0.001	−0.08
	Unemployed	42 (23)			
Psychological treatment	Yes	36 (26)	813,396.50	<0.001	−0.06
	No	40 (24)			
Medications	Yes	43 (25)	1045,481.50	<0.001	−0.06
	No	39 (24)			
Suicide attempt	Yes	54 (19)	224,144.00	<0.001	−0.14
	No	39 (24)			
Educational level	≤ 12 years	44 (22)	1979,192.00	<0.001	−0.12
	≥13 years	38 (25)			
Time since loss	<6 months	40 (24)	1529,027.50	<0.001	−0.07
	≥6 months	36 (27)			

*Mdn, Median; IQR, interquartile range; r, Rosenthal's r*.

Regarding the impact of time since the loss of the loved one, participants whose loss was more recent (<6 months) showed higher levels of depression (*p* < 0.001), sleep difficulties (*p* < 0.01), and problematic grief symptoms (*p* < 0.001), compared to respondents whose loss occurred 6 months ago or more. On the contrary, individuals whose loss occurred more recently (<6 months prior) reported lower levels of arousal than participants with a loss that occurred 6 months ago or more. No significant differences were found for anxiety and avoidance when comparing the participants considering time since the loss (Supplementary Tables 1–5).

### Correlates of Anxiety Severity

Table 3 summarizes the comparisons between anxiety levels (none, mild, moderate, and severe anxiety) and their correlates. Chi-square analyses were significant for all binary variables, except for psychological support and for time since loss. That is, gender, age, working status, educational level, use of medication, and recent suicide attempt were associated with anxiety.

**Table 3:** Associations between anxiety levels and predictor variables.

	**Anxiety symptoms**										
	**No (*****n*** **=** **329)**		**Mild (*****n*** **=** **1,097)**		**Moderate (*****n*** **=** **1,533)**		**Severe (*****n*** **=** **2,265)**				
**Predictors**	***N***	**%**	***n***	**%**	***n***	**%**	***n***	**%**	***X* ^2^**	***p***	**Cramer's V**
Gender									37.07	<0.001	0.084^**^
Female	266	5.8	926	20.3	1,329	29.2	2,038	44.7			
Male	63	9.6	169	25.8	200	30.6	222	33.9			
Total	329	6.3	1,095	21.0	1,529	29.3	2,260	43.4			
Age ^†^									46.48	<0.001	0.094^**^
≤ 30 years old	114	4.7	440	18.3	724	30.1	1,128	46.9			
≥31 years old	215	7.6	657	23.3	809	28.7	1,137	40.3			
Total	329	6.3	1,097	21.0	1,533	29.3	2,265	43.4			
Working									17.91	<0.001	0.059^**^
Yes	212	6.8	701	22.6	886	28.6	1,301	42.0			
No	117	5.5	396	18.6	647	30.5	964	45.4			
Total	329	6.3	1,097	21.0	1,533	29.3	2,265	43.4			
Education									11.07	0.011	0.046^*^
≤ 12 years	66	5.3	232	18.7	364	29.3	581	46.7			
≥13 years	263	6.6	865	21.7	1,169	29.4	1,684	42.3			
Total	329	6.3	1,097	21.0	1,533	29.3	2,265	43.4			
Psychological support									2.20	0.534	–
Yes	27	6.7	88	21.9	105	26.1	182	45.3			
No	295	6.3	968	20.7	1,382	29.6	2,021	43.3			
Total	322	6.4	1,056	20.8	1,487	29.3	2,203	43.5			
Medication									82.21	<0.001	0.127^**^
Yes	10	1.9	59	11.5	133	25.9	311	60.6			
No	312	6.8	997	21.9	1,354	29.7	1,892	41.5			
Total	322	6.4	1,056	20.8	1,487	29.3	2,203	43.5			
Recent suicide attempt									56.37	<0.001	0.105^**^
Yes	1	0.6	10	6.1	38	23.2	115	70.1			
No	321	6.5	1,046	21.3	1,449	29.5	2,088	42.6			
Total	322	6.4	1,056	20.8	1,487	29.3	2,203	43.5			
Time since loss									0.04	0.998	–
≥6 months	51	6.3	166	20.6	238	29.6	349	43.4			
<6 months	271	6.4	890	20.9	1,249	29.3	1,854	43.5			
Total	322	6.4	1,056	20.8	1,487	29.3	2,203	43.5			
	Mdn	IQR	Mdn	IQR	Mdn	IQR	Mdn	IQR	*H*	*p*	${\text{E}}_{\text{R}}^{2}$
Age^†^	35.00	16.00	34.00	16.00	31.00	15.00	31.00	13.00	44.047	<0.001	0.008
Depression	13.00	12.50	21.00	16.00	31.00	17.50	42.00	16.00	1,431.690	<0.001	0.274
Sleep problems	8.00	5.00	10.00	5.00	12.00	4.00	14.00	5.00	661.118	<0.001	0.127
Avoidance	4.00	6.00	7.00	6.00	10.00	6.00	14.00	7.00	1,123.558	<0.001	0.215
Activation	3.00	3.00	5.00	4.00	8.00	4.00	11.00	4.00	1,791.972	<0.001	0.343
Grief symptoms	18.00	18.00	28.00	21.00	39.00	19.00	49.00	20.00	1,295.775	<0.001	0.254

† *The age variable is shown as a quantitative and categorical*.

* *p < 0.05;*

** *p < 0.001*.

Kruskal-Wallis tests showed that the more severe the anxiety, the higher the levels of depression, sleep difficulties, avoidance, arousal, and grief symptoms. H values were statistically significant (all *p* < 0.001).

Regarding age, a different trend was observed: the more severe the anxiety, the lower the mean age of the participants. The H value was statistically significant (*p* < 0.001).

Categorical variables with significant *p*- values were included in the multinomial logistic regression.

Next, a multinomial logistic regression was undertaken to examine the extent to which individual variables remained significant predictors of anxiety accounting for shared variance among predictors. Age was considered a binary variable for a more comprehensive interpretation. The reference category was the non-anxiety group. Each level (*mild, moderate, severe*) was compared with this reference category. The multinomial logistic regressions and adjusted odd ratios were calculated and are shown in Table 4.

**Table 4:** Multinomial logistic regression for anxiety.

	**Adjusted odds ratio**					
	**Mild**		**Moderate**		**Severe**	
**Predictors**	**OR (95% CI)**	***p***	**OR (95% CI)**	***p***	**OR (95% CI)**	***p***
**Gender**						
Female	1.34 (0.97–1.85)	0.079	1.60 (1.16–2.19)	0.004	2.18 (1.59–2.99)	<0.001
**Age**						
≤ 30 years old	1.31 (1.00–1.70)	0.049	1.70 (1.32–2.19)	<0.001	1.90 (1.48–2.44)	<0.001
**Working**						
No	0.93 (0.71–1.21)	0.571	1.15 (0.89–1.49)	0.292	1.11 (0.86–1.44)	0.411
**Education**						
≤ 12 years	1.11 (0.81–1.53)	0.514	1.23 (0.90–1.68)	0.189	1.35 (1.00–1.83)	0.052
**Drugs**						
Yes	1.92 (0.97–3.80)	0.063	3.32 (1.72–6.40)	<0.001	5.64 (2.96–10.73)	<0.001
**Recent suicide attempt**						
Yes	2.90 (0.37–22.76)	0.312	7.20 (0.98–52.77)	0.052	14.93 (2.07–107.67)	0.007

The final model had a−2 Log Likelihood value of 499.560, χ^2^ (18, N = 5,056) =249.952, *p* < 0.001. The Nagelkerke Pseudo R-Square was 0.053, which suggested that ~5.3% of the variance associated with anxiety levels was explained by the predictors.

Moderate and severe anxiety groups exhibited significant associations with gender, age, and substance intake. The influence of these variables was strong. Regarding gender, women were 1.6–2.2 times more likely than men to exhibit problematic anxiety symptoms, adjusting for the rest of the variables. Younger participants were 1.7–1.9 times more likely to be in these anxiety groups (vs. the reference group) than older ones, when controlling for the other predictors. Comparatively, participants who used medication had a risk of 3.3–5.6 times greater of being in the moderate anxiety and severe anxiety groups (vs. the reference group) compared to those who did not use substances, even when controlling for other independent variables. In addition, the severe anxiety group also showed an association with recent suicide attempts: participants had almost 15 times more risk of exhibiting these manifestations of anxiety than people without a recent suicide attempt. On the other hand, working status and level of education were not significant predictors of anxiety severity.

As mentioned in previous paragraphs, we reported the associations between anxiety and medication use, the latter presented in a dichotomous variable format (yes/no). Next, the type of medication and the connection with anxiety levels are explored and shown in Table 5 (Supplementary Table 6 presented international non-proprietary names for the substances, also the number and percentage of users for each of them).

**Table 5:** Medication use and types and their relationship to anxiety.

		**Anxiety symptoms**					
**Medication Type**	**Users**	**No**	**Mild**	**Moderate**	**Severe**		
	***n***	***n***	***n***	***n***	***n***	***X^2^***	***P***
**Antidepressants**							
SSRI						0.54	0.910
Yes	239	4	29	60	146		
No	274	6	30	74	164		
Other						2.21	0.530
Yes	61	0	6	19	36		
No	452	10	53	115	274		
**Anxiolytics**							
BZD						18.67	<0.001
Yes	212	3	13	46	150		
No	301	7	46	88	160		
Other						1.33	0.723
Yes	24	0	4	7	13		
No	489	10	55	127	297		
Antipsychotics						2.36	0.491
Yes	56	0	5	13	38		
No	457	10	54	121	272		
Anticonvulsant						1.54	0.700
Yes	39	0	6	9	24		
No	474	10	53	125	286		
Natural, naturist, homeopathic						3.20	0.362
Yes	46	1	4	17	24		
No	467	9	55	117	286		
Other substances						6.26	0.095
Yes	11	1	0	5	5		
No	502	9	59	129	305		
Unspecified						8.13	0.067
Yes	19	2	1	5	11		
No	494	8	58	129	299		

From the total sample (*N* = 5,224), 513 (9.80%) individuals informed the use of medication. A total of 354 (6.8%) of the respondents reported consuming more than one substance. Antidepressants were the most frequently reported medication. In this category, selective serotonin reuptake inhibitors (SSRIs) were the most frequently used, with 233 (4.5%) participants using one of them and 6 (0.1%) subjects taking two of these substances. Anxiolytics were the second group of medicines most frequently mentioned; within this type, benzodiazepines (BZDs) were the most common with 209 (4.0%) participants reporting the use of one kind of this medication, and 3 (0.1%) respondents who acknowledged taking two different kinds of them.

Chi-square tests presented a significant association between anxiety and BZD (*p* < 0.001). Cramer's V was 0.191 (*p* < 0.001), which suggested that the two variables shared about 3.6% of their variance. The adjusted residual for participants with more severe anxiety presentation was 4.0 (>1.96 in absolute value), indicating that for respondents of this group who were taking BZD, and observed frequencies (*n* = 150) were higher than expected (*n* = 128). None of the other types of drugs showed significant relationships with anxiety.

## Discussion

The main objective of this study was to examine the prevalence of anxiety symptoms and associated clinical and sociodemographic factors in Mexican adults seeking psychological support for grief during the COVID-19 pandemic. The results showed that 93.7% of participants exhibited significant anxiety symptoms, 95.2% had sleep problems, 86.6% presented symptoms of depression, and 67.7% showed potentially problematic grief symptoms.

The literature currently available regarding the mental health impact caused by COVID-19 is accumulating, and information from low-income and developing countries is starting to appear, but at a considerably lower rate than in developed countries. A part of the population that is potentially suffering more severely than the general population due to the ongoing pandemic is represented by the people that have been bereaved during the COVID-19 pandemic, where funerals and other important rituals were not allowed to take place. Regarding this group, Eisma et al. (33) warned that prolonged grief disorder will be a public health problem once the COVID-19 pandemic ends. Circumstances of death during the pandemic are likely to increase the prevalence of a prolonged grief disorder, such as multiple and indirect traumatic characteristics, such as possible multiple deaths for families that could lead to bereavement overload, due to limitations caused by COVID-19 that restrict families from spending the last days together with the person they lost and from performing on-site funerals (16). Other authors suggest that further research is necessary on the impact of COVID-19 on grievers.

The results showed that being younger was associated with greater symptomatology in anxiety, depression, grief, avoidance, and arousal. Individuals younger than 30 had more severe symptoms than those older than 30. In previous research, it has been observed that young adults may engage in negative thinking as a coping strategy, which would bring about maladaptive outcomes (34). Importantly, older adults tend to have more stable jobs with better salaries (35), which could act as a protective factor.

In addition, the present study shows a high prevalence of posttraumatic stress related symptoms. These symptoms were higher for females than for males. Also, unemployed people presented more avoidance and arousal symptoms than employed ones, highlighting this group as one of the most affected by the pandemic. This supports prior research showing that unemployment is directly linked to insecurity about work and finances and worse mental health in terms of anxiety and depression (35). It has also been observed that during the COVID-19 pandemic, women were 24% more likely to permanently lose their jobs than men. In addition, women expected their labor income to fall 50% more than men (36). Also, considering the available literature and the results of the present study, there is evidence that during the COVID-19 pandemic, women are more affected than men in terms of mental health. The reasons could be several, such as domestic violence, parenting, miscarriage, pregnancy, job loss, or postpartum depression (37, 38).

Furthermore, attempt of suicide in the last 3 months had significantly greater symptomatology or impact in all the areas measured in this study (anxiety, depression, sleep quality, grief, avoidance, arousal, and medications consumed), compared to the participants that had not recently attempted suicide. These results are in accordance with previous studies. A study conducted with middle-aged and older adults with depressive symptoms in five low- and middle-income countries, concluded that the participants with poor or very poor sleep quality had a greater likelihood of suicidal ideation, and those with moderate and severe or extreme insomnia had a greater likelihood of suicidal ideation and suicidal attempt (39). Other studies identified that anxiety is a statistically yet weak predictor of suicide ideation and attempts but not deaths (40). Another study has observed that suicide attempters and completers were similar in terms of depression, but suicide completers were more likely to use alcohol or medication (41). The results of our study add to the growing literature on mental health outcomes following bereavement during the pandemic. Future studies will assist in establishing the generalizability of our findings. On the other hand, time since loss was also relevant, indicating that people with a more recent loss were more affected in grief areas than those who reported more time since their loss. Additionally, the participants whose loss was <6 months prior to the assessment showed higher levels of depression and of sleep difficulties compared to those whose loss occurred 6 months or more prior to their access to the platform. However, individuals whose loss occurred <6 months ago reported lower levels of arousal compared to participants with a loss that occurred 6 months or more before their first access to the platform. This result reflects the need for future longitudinal studies to understand the trajectory of normal grieving and its relationships with post-traumatic stress symptoms over time. Whereas, studies with participants diagnosed with prolonged grief disorder have shown associations between this disorder and post-traumatic symptoms, including hyperarousal (42), the link between normal grieving and arousal trajectory, considering time since loss, is still scarce. More evidence is required considering the causes of death (natural vs. unnatural causes), the presence of a traumatic vs. a non-traumatic loss (43), and the closeness with the deceased person.

No significant differences were found for anxiety and avoidance when comparing the participants on time since loss. These results may suggest that, concerning the time elapsed since the loss, even after 6 months, people continue to display indicators of emotional distress, specifically posttraumatic stress symptoms (avoidance and arousal) that, in turn, are associated with high levels of anxiety. This is consistent with the presence of depressive and post-traumatic symptoms that have been reported as frequent among people who have experienced a recent loss (44).

Furthermore, researchers worldwide are concerned about the impact of the circumstances of the COVID-19 pandemic and the possible rise in the prevalence of prolonged grief disorder (45). In this sense, a recent study (46) compared the grief symptomatology in participants that had lost someone due to COVID-19 with those who lost someone due to natural causes, such as other illnesses or old age, and unnatural causes, such as accidents, suicide, or homicide. The researchers observed that, as a result of COVID-19, grievers reported more severe symptoms of prolonged grief disorder and persistent complex bereavement disorder compared to natural bereavement, but not to unnatural bereavement. Likewise, the study of Eisma and Tamminga (47) compared the results of individuals who lost someone before and during the pandemic, observing that in general, grief severity was not significantly different; nevertheless, the people that experienced a recent loss (according to the authors 5 months ago and less) during the pandemic had higher grief levels compared to a similar experience not during the pandemic. These results could suggest that a more recent loss in terms of time is related to higher symptomatology during the COVID-19 pandemic. Another study with recently bereaved spouses observed considerable somatic symptoms during the earliest months of the loss but there were no major health declines over the first year and a half due to the grief symptoms (48). Similarly, Tang and Xiang (49) reported that in a sample of 422 participants who had lost someone due to COVID-19, the prevalence of prolonged grief disorder was between 29.3 and 37.8%, depending on the screening tool.

It is relevant to notice that the vast majority of the participants in this study indicated reduced sleep quality. The sleep quality was significantly lower for older adult participants. Also, analyzing the relationship between the time since loss and sleep quality, significant differences were found (difficulties were greater for those whose loss was more recent). This is in line with the data of other developing countries like Mexico, such as India, where it has been observed that the lockdown was associated with poor sleep quality and shifts in sleep cycles (50). Data from other countries, such as Portugal, indicated that from 365 participants, two-thirds reported at least one sleep difficulty and frequent awakenings (51). To the best of our knowledge, this is the first study that presents data related to sleep quality of bereaved individuals during the COVID-19 pandemic. The research related to sleep disturbances of people suffering from complicated grief is still scarce. It has been observed that in community-dwelling middle-aged and older adults, persons with normal and complicated grief had both a shorter sleep duration and a lower sleep quality, mainly explained by depressive symptoms (52). In the study of Szuhany et al. (53) with 395 patients with complicated grief, greater complicated grief was associated with poorer sleep quality. More research is needed in this line and it is expected that this manuscript provides more data in this line.

Regarding medication consumption, the main medications used were SSRIs, followed by BZDs. A total of 513 individuals reported having consumed medication, and of those, more than half acknowledged taking more than one. This is a considerable proportion, and further studies should be conducted to gain a better understanding of using medication as a coping strategy related to the COVID-19 pandemic. Furthermore, it has been widely observed that alcohol consumption has increased during the COVID-19 pandemic (54), although the WHO discourages the use of substances with the potential to create addiction to manage the burden of social isolation (55), BZDs are often the first-line pharmacological treatment for various anxiety disorders such as General Anxiety Disorder and Social Anxiety Disorder, among others (56). Although there is no confirmed diagnosis of the participants of this study, it was found that severe anxiety symptoms are present in a large part of the sample, symptoms that, due to the significant association found with BZD, are probably being addressed through these medications. These findings give way to a reflection on possible difficulties derived from its use, especially in the medium or long term. This is an aspect that should be included in future studies.

Despite the limitation of the cross-sectional design used in this study, the results presented are relevant because they reveal the high rate of symptoms in bereaved people during the pandemic, specifically the levels of anxiety, depression, affected sleep quality, avoidance and arousal, complicated grief symptoms, and medication consumption. The present results are in line with other studies that have used a cross-sectional design during the COVID-19 pandemic (57, 58). It is recommended that future studies focus on evaluating the trajectory of the grief symptoms of people that lost someone during the COVID-19 pandemic.

Another limitation to the current study was the lack of a comparison group to evaluate the difference in clinical indicators between those who were bereaved and those who were not bereaved during the pandemic. Therefore, the results should be received with caution.

A further limitation is that this study had a considerably a greater sample of women, compared to men. This is in accordance with many other studies where women are less reluctant to search for psychological support than men (59). Also, it has been observed that the prominent obstacles for men to search for psychological help are disinclination to express emotions or concerns about health, embarrassment, anxiety, fear, and poor communication with health professionals, which applies not only to psychological support but also to physical health (60). Another reason could be that men's ideas regarding masculinity are a considerable barrier to seek psychological support (59). It is of interest to researchers that these stereotypes persist even in such a stressful time for humankind, such as this global pandemic. Future studies should make more efforts to include more men in the samples by creating advertisements indicating that it is positive and acceptable for men to ask for psychological support.

Moreover, it must be mentioned that there was no data collected on the cause of death. This information could have been useful in teasing out the differences in anxiety (and associated factors) based on loss due to COVID-19 vs. other causes. Other relevant variables could be added, such as socioeconomic status (61) or social connections (62) of the responder, among other variables, which could influence the psychological impact of the loss. In addition, the sample included only those who sought psychological help. Regarding the strengths of the study, several can be mentioned. This is one of the first studies that has presented the symptomatology of anxiety, depression, sleep quality, avoidance, arousal, grief, and medication consumption in a sample of people that are in a grieving process and actively seeking psychological support due to the loss of someone during the COVID-19 pandemic. Part of this strength is that the sample size was considerably large, with 5,224 participants. Other studies in similar developing countries could be considered in order to further explore the impact of the pandemic on grief.

## Conclusion

In conclusion, with these results, it has been observed that the most affected sector of the Mexican population with symptoms of anxiety, depression, avoidance, arousal, and grief are unemployed young women that consume medication and have attempted suicide in the last 3 months.

A more recent loss (<6 months prior to participating in the study) was significantly associated with higher levels of depression, sleep difficulties, and grief symptoms, and with lower levels of post-traumatic arousal symptoms, when compared to a loss that occurred 6 months ago or more.

These results indicate the need to provide mental health treatment to the population that suffered the death of a loved one during the COVID-19 pandemic to reduce the impact in terms of mental health, even when the pandemic is under control. In addition, the results of the efficacy of the intervention *Duelo COVID* will be presented in upcoming manuscripts.

## Data Availability Statement

Original datasets are available in a publicly accessible repository: The original contributions presented in the study are publicly available. This data can be found here: https://www.datafirst.uct.ac.za/dataportal/index.php/catalog/865.

## Ethics Statement

The study design and procedures were approved by the Research Ethics Committee of the Autonomous University of Ciudad Juárez, México (Approval ID: CEI-2020-2-226) and are registered in Clinical trials (NCT04638842). The patients/participants provided their written informed consent to participate in this study.

## Author Contributions

AD-R, PH-A, JM, EE, and LF accessed and verified the underlying data and take responsibility for the integrity of the data and accuracy of the corresponding analysis. AD-R, AR-G, MH, EB, RC, CA-S, PA-L, and SM-L conceptualized the study. PH-A and LF maintained data over time and linked data across data collection waves over time. PH-A, JM, EE, and LF did the formal analyses. AD-R and FR-M acquired the funding for this study. AD-R was the project administrator and supervisor. RM, EB, and JM conducted the literature review and organization of the manuscript. All authors wrote the original manuscript draft, contributed to reviewing, editing the manuscript, had full access to all the data, and had ultimate responsibility for the decision to submit the manuscript for publication.

## Funding

This research was funded by the Universidad Autónoma de Ciudad Juárez (UACJ). The University had no role in any steps of the research: study design, collection, analysis, and interpretation of data, the writing of the manuscript, or the decision to submit the paper for publication. Financial aid for the publication was granted by the Universidad Internacional del Ecuador (UIDE).

## Conflict of Interest

The authors declare that the research was conducted in the absence of any commercial or financial relationships that could be construed as a potential conflict of interest.

## Publisher's Note

All claims expressed in this article are solely those of the authors and do not necessarily represent those of their affiliated organizations, or those of the publisher, the editors and the reviewers. Any product that may be evaluated in this article, or claim that may be made by its manufacturer, is not guaranteed or endorsed by the publisher.
