# Supplementary material for: Prevalence of Anxiety Symptoms and Associated Clinical and Sociodemographic Factors in Mexican Adults Seeking Psychological Support for Grief During the COVID-19 Pandemic: A Cross-Sectional Study
Source: Front Psychiatry. 2022 Mar 14;13:749236. doi: 10.3389/fpsyt.2022.749236 (PMC8964437; doi:10.3389/fpsyt.2022.749236)
Supplement: Supplementary file 1 [file Data_Sheet_1.pdf]

Supplementary Material

Table 1. Normality and homoscedasticity testing for dependent variables according to sociodemographic dimensions

	Anxiety		Depression		Sleep		Avoidance		Activation	
(n; %)	K-W	Levene	K-W	Levene	K-W	Levene	K-W	Levene	K-W	Levene
Gender										
Female (4559; 87.3)	<0.001	0.102	<0.001	0.106	<0.001	0.669	<0.001	0.507	<0.001	0.150
Male (654; 12.5)	<0.001		<0.001		<0.001		<0.001		<0.001	
Age in years										
≤ 30 (2406; 46.1)	<0.001	<0.001	<0.001	<0.001	<0.001	0.009	<0.001	<0.001	<0.001	0.028
≥ 31 (2818; 53.9)	<0.001		<0.001		<0.001		<0.001		<0.001	
Working										
Yes (3100; 59.3)	<0.001	0.003	<0.001	0.074	<0.001	0.950	<0.001	<0.001	<0.001	0.361
No (2124; 40.7)	<0.001		<0.001		<0.001		<0.001		<0.001	
Education in years										
≤ 12 (1243;23.8)	<0.001	0.198	<0.001	0.075	<0.001	0.335	<0.001	0.070	<0.001	0.981
≥ 13 (3981;76.2)	<0.001		<0.001		<0.001		<0.001		<0.001	
Psychological support										
Yes (402; 7.7)	<0.001	0.352	0.001	0.137	<0.001	0.001	0.001	0.192	<0.001	0.074
No (4666; 89.3)	<0.001		<0.001		<0.001		<0.001		<0.001	
Medications										
Yes (513; 9.8)	<0.001	<0.001	<0.001	0.066	<0.001	0.250	<0.001	0.952	<0.001	0.715
No (4555; 87.2)	<0.001		<0.001		<0.001		<0.001		<0.001	
Suicide attempt										
Yes (164; 3.14)	<0.001	<0.001	<0.001	<0.001	<0.001	0.061	<0.001	<0.001	<0.001	0.019
No (4904; 93.9)	<0.001		<0.001		<0.001		<0.001		<0.001	
Time since loss										
<6 months (4264; 84.6)	<0.001	0.904	<0.001	0.072	<0.001	0.301	<0.001	0.001	<0.001	0.127
≥6 months (804; 15.4)	<0.001		<0.001		<0.001		<0.001		<0.001	

Table 2. Normality and homoscedasticity testing for dependent variables according to anxiety symptoms severity

	Depression		Sleep		Avoidance		Activation	
(n; %)	K-W	Levene	K-W	Levene	K-W	Levene	K-W	Levene
Anxiety Symptoms								
No (329; 6.3)	<0.001	0.042	<0.001	0.121	<0.001	0.024	<0.001	<0.001
Mild (1097; 21.0)	<0.001		<0.001		<0.001		<0.001	
Moderate (1533; 29.3)	<0.001		<0.001		<0.001		<0.001	
Severe (2265; 43.4)	<0.001		<0.001		<0.001		<0.001	

Table 3. Missing data for predictor variables

Predictor variable	Complete cases, n (%)	Missing cases, n (%)	χ^2	*p*-value
Psychological treatment/ Pharmacological Treatment/ Suicide attempt in the last three months/ Time since loss	5068/5224 (97·01%)	156/5224 (2.99%)	3.46	.325
Gender	5213/5224 (99.79%)	11/5224 (0.21%)	0.93	.866

Note. The χ^2 value (4X2 Tables) explored whether the outcome variable anxiety (no, mild, moderate, severe) was associated with the predictors comparing complete and not complete data.

Table 4. Intercorrelation matrix among clinical variables

	1.	2.	3.	4.	5.	6.
1. Anxiety	-					
2. Depression	0.636*	-				
3. Sleep problems	0.480*	0.519*	-			
4. Avoidance	0.574*	0.617*	0.418*	-		
5. Activation	0.709*	0.600*	0.541*	0.640*	-	
6. Grief	0.539*	0.649*	0.385*	0.647*	0.520*	-
Mdn	13.00	33.00	12.00	11.00	8.00	40.00
IQR	9.00	22.00	6.00	8.00	6.00	24.00

Note. *p <0.001

Table 5. Time since loss and anxiety: Exploring time variable in depth

		Anxiety symptoms				
Time since loss		No	Mild	Moderate	Severe	Total
Less than a month	Count (%)	124 (5.9%)	447 (21.2%)	560 (26.6%)	975 (46.3%)	2106
	E.C.	133.8	438.8	617.9	915.5	
	A. R.	-1.1	0.6	-3.6	3.4	
One month	Count (%)	61 (7.3%)	151 (18.2%)	262 (31.5%)	357 (43.0%)	831
	E.C.	52.8	173.2	243.8	361.2	
	A. R.	1.3	-2.1	1.5	-0.3	
Two months	Count (%)	33 (6.6%)	125 (25.2%)	137 (27.6%)	202 (40.6%)	497
	E.C.	31.6	103.6	145.8	216.0	
	A. R.	0.3	2.5	-0.9	-1.3	
Three months	Count (%)	21 (6.4%)	62 (19.0%)	118 (36.2%)	125 (38.3%)	326
	E.C.	20.7	67.9	95.7	141.7	
	A. R.	0.1	-0.8	2.8	-1.9	
Four months	Count (%)	14 (6.7%)	40 (19.0%)	68 (32.4%)	88 (41.9%)	210
	E.C.	13.3	43.8	61.6	91.3	
	A. R.	0.2	-0.7	1.0	-0.5	
Five months	Count (%)	18 (6.1%)	65 (22.1%)	104 (35.4%)	107 (36.4%)	294
	E.C.	18.7	61.3	86.3	127.8	
	A. R.	-0.2	0.6	2.3	-2.5	
Six months	Count (%)	19 (5.7%)	54 (16.3%)	109 (32.9%)	149 (45.0%)	331
	E.C.	21.0	69.0	97.1	143.9	
	A. R.	-0.5	-2.1	1.5	0.6	
More than six months ago	Count (%)	32 (6.8%)	112 (23.7%)	129 (27.3%)	200 (42.3%)	473
	E.C.	30.1	98.6	138.8	205.6	
	A. R.	0.4	1.6	-1.0	-0.5	
Total	Count (%)	322 (6.4%)	1056 (20.8%)	1487 (29.3%)	2203 (43.5%)	5068

Note. E.C= Expected count. A.C=Adjusted residual. χ^2= 45.62, $p< 0.001$. Cramer´s V= 0.095, $p< 0.001$

Table 6. List of medication reported by participants

Medication type	Active principle	n	%
Antidepressants			
SSRIs			
	Citalopram	19	0.4
	Escitalopram	37	0.7
	Fluoxetine	85	1.6
	Fluvoxamine	2	0.0
	Paroxetine	34	0.7
	Sertraline	68	1.3
Other			
	Amitriptyline	12	0.2
	Desvenlafaxine	4	0.1
	Duloxetine	12	0.2
	Imipramine	8	0.2
	Mirtazapine	5	0.1
	Trazodone	1	0.0
	Venlafaxine	18	0.3
	Vortioxetine	1	0.0
Anxiolytics			
BZD			
	Alprazolam	45	0.9
	Bromazepam	6	0.1
	Clobazam	1	0.0
	Clonazepam	135	2.6
	Diazepam	20	0.4
	Estazolam	2	0.0
	Ethyl Loflazepate	2	0.0
	Lorazepam	3	0.1
	Triazolam	1	0.0
Other			
	Buspirone	1	0.0
	Hydroxyzine	23	0.4
Antipsychotics			
	Amisulpride	1	0.0
	Aripiprazole	2	0.0
	Haloperidol	2	0.0
	Lithium carbonate	1	0.0
	Olanzapine	4	0.1
	Perphenazine	10	0.2
	Quetiapine	29	0.6
	Risperidone	6	0.1
	Sulpiride	1	0.0

Table 6. List of medication used by participants (continuation)

Medication type	Active principle	n	%
Anticonvulsant medication			
	Carbamazepine	11	0.2
	Gabapentin	2	0.0
	Lamotrigine	2	0.0
	Levetiracetam	2	0.0
	Magnesium Valproate	8	0.2
	Phenytoin	3	0.1
	Pregabalin	9	0.2
	Topiramate	3	0.1
Natural, naturist, or Homeopathic substances			
	Cannabis	8	0.2
	Homeopathic	7	0.1
	Lavandulae aetheroleum	1	0.0
	Lemon balm	6	0.1
	Melatonin	7	0.1
	Passiflora	5	0.1
	Valerian	16	0.3
	Vitamins/ naturists	7	0.1
Other substances			
	Atomoxetine	1	0.0
	Bisoprolol	1	0.0
	Buprenorphine patches	1	0.0
	Cinnarizine	1	0.0
	Diphenhydramine	1	0.0
	Flunarizine	1	0.0
	Methylphenidate	1	0.0
	Ofloxacin	1	0.0
	Propranolol	1	0.0
	Tramadol	1	0.0
	Zolpidem	1	0.0
*Unspecified		19	0.3

Note. SSRI= Selective serotonin reuptake inhibitors; BZD= Benzodiazepines; *Unspecified category included information expressed by participants as “Antidepressant” (n= 5; 0.1%), “Anxiolytic” (n= 8; 0.2%) or “For sleep” (n=6; 0.1%).
